# Supplementary figures and images for: Human umbilical cord blood monocytes, but not adult blood monocytes, rescue brain cells from hypoxic-ischemic injury: Mechanistic and therapeutic implications
Source: PLoS One. 2019 Sep 4;14(9):e0218906. doi: 10.1371/journal.pone.0218906 (PMC6726370; doi:10.1371/journal.pone.0218906)

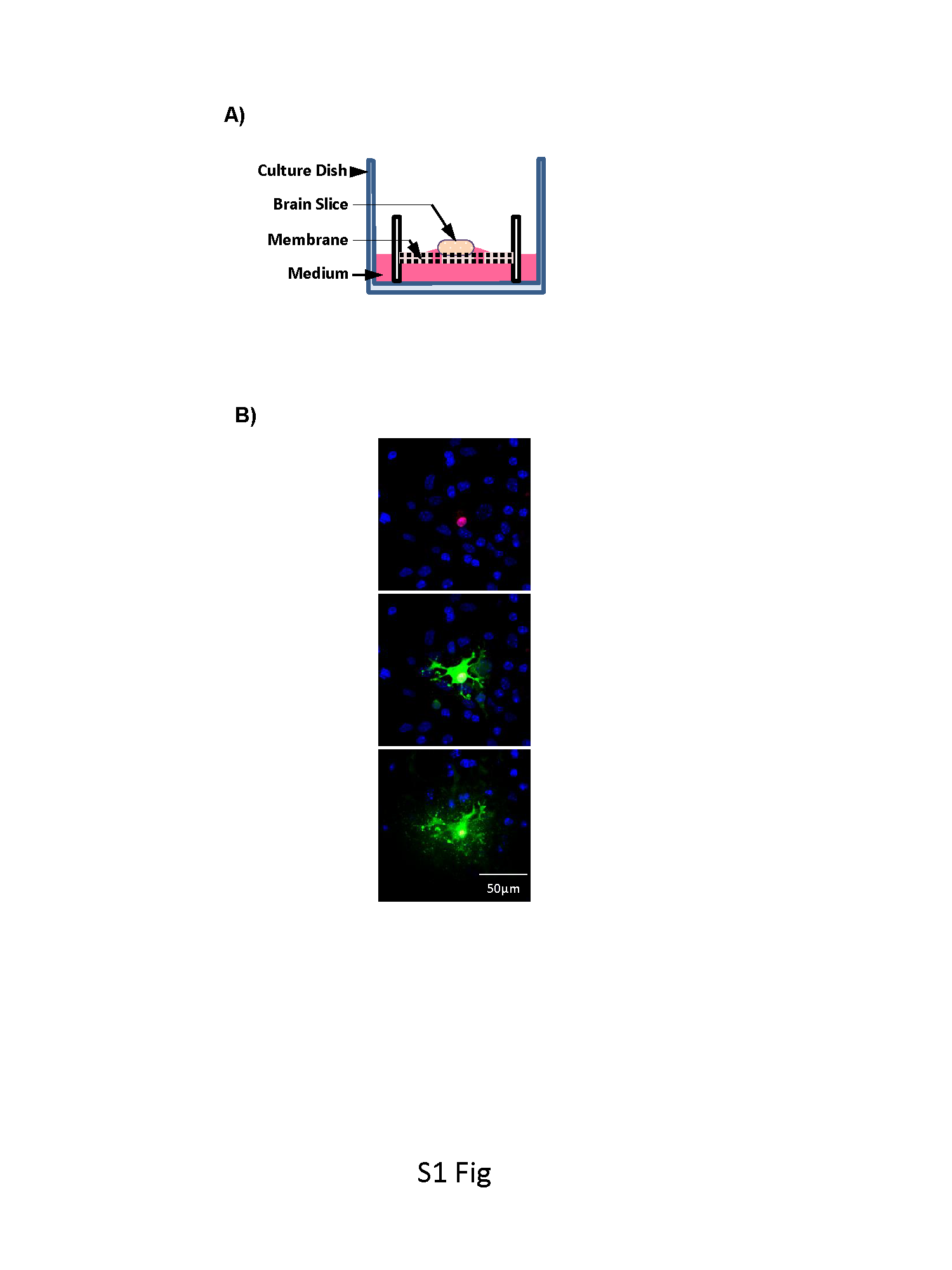

Supplement: S1 Fig — (TIF) [file pone.0218906.s001.tif]

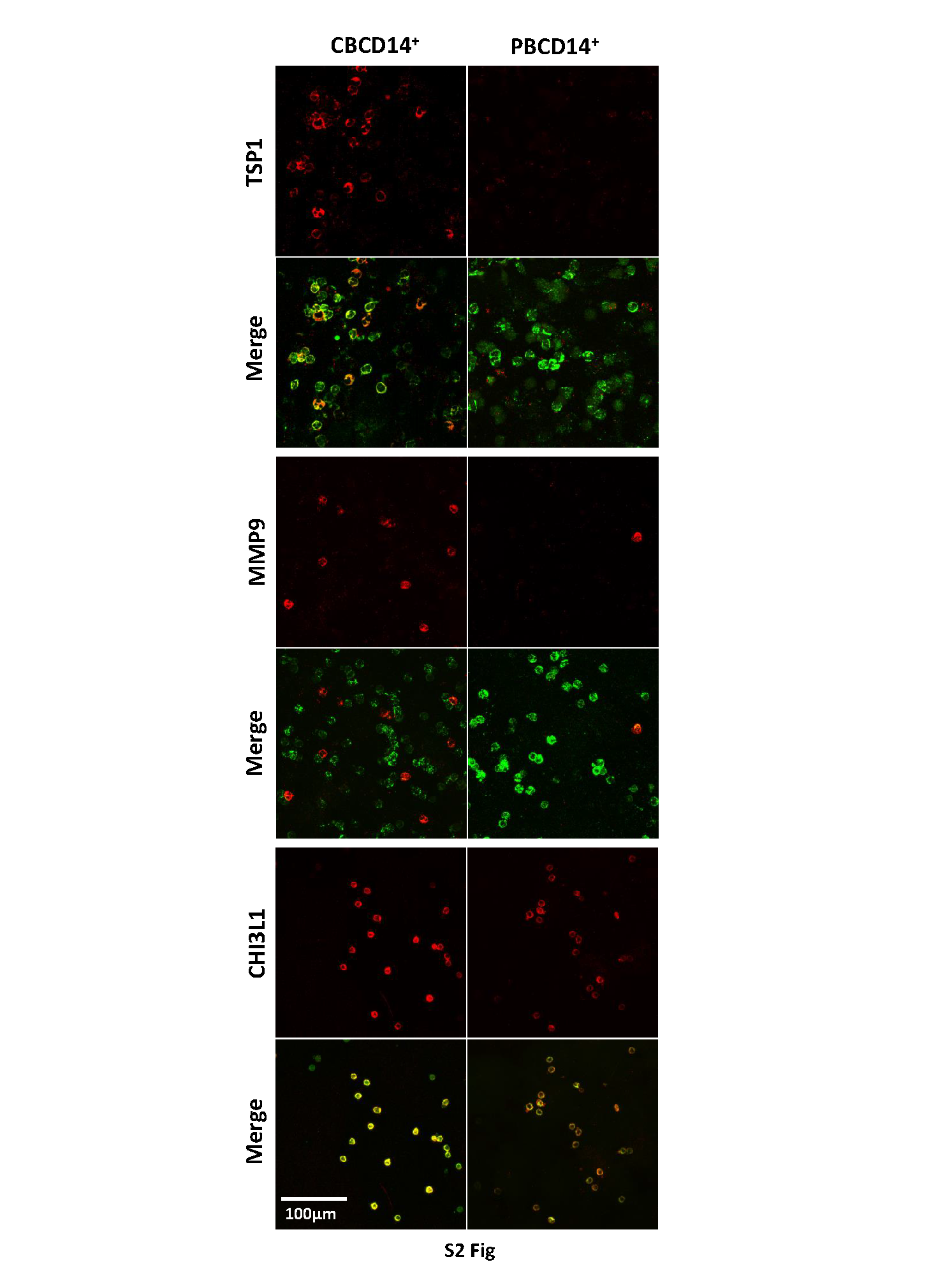

Supplement: S2 Fig — (TIF) [file pone.0218906.s002.tif]

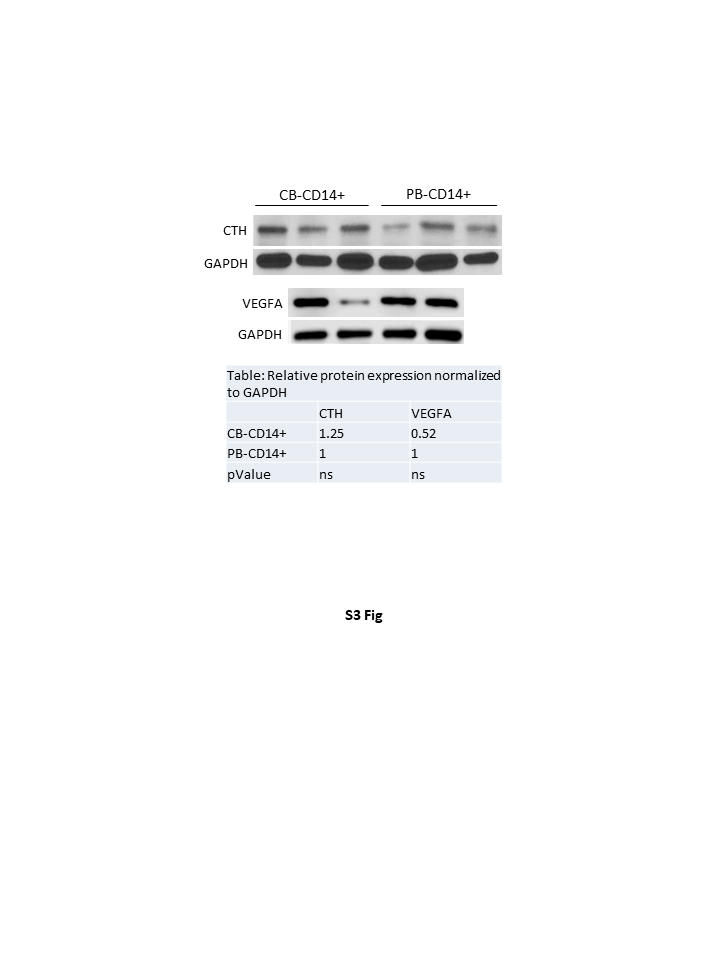

Supplement: S3 Fig — (TIF) [file pone.0218906.s003.TIF]
