# Supplementary material for: Human umbilical cord blood monocytes, but not adult blood monocytes, rescue brain cells from hypoxic-ischemic injury: Mechanistic and therapeutic implications
Source: PLoS One. 2019 Sep 4;14(9):e0218906. doi: 10.1371/journal.pone.0218906 (PMC6726370; doi:10.1371/journal.pone.0218906)
Supplement: S1 File — (DOCX) [file pone.0218906.s004.docx]

**Supporting Information**

**Method:**

**Carboxyfluorescein succinimidyl ester (CFSE) labeling**

In order to detect human cells in mouse brain slice culture , human CB-MNC were stained with 5μM CFSE, V12883, green fluorescence (Life Technologies)prior to addition to brain slices as described by the manufacturer to track cells[1]. Labeled cells were incubated with OGD-shocked slices for 72 hours under the standard conditions used for neuroprotection assays. Cultures were then fixed , counter stained with human specific antibody (HuN, Abcam, 1:100), which stains the human cell nuclei to confirm that cells showing green CFSE fluorescence in the brain tissue were of human origin.

**Microarray analysis:** S3 Table gives full MS5 analysis for the two chips comparing CB and PB monocyte gene expression. This analysis simply scores the normalized fluorescence signal for each probe set as expressed or not expressed compared to background. In both experiments 714 and 1213 in which CB and PB cells were compared about 18,500 probe sets detected transcripts expressed in all CB samples (line 1, S3 Table), about 20,000 probe sets detected transcripts expressed by all PB samples (line 3), and about 24,000 probe sets did not detected expressed transcripts in either cell population (line 5). Thus, there was reasonable agreement in these gross expression parameters between the two analyses. As expected, there was more variability in the number of probes detecting mixed expression (expression in only 1 or 2 of 3 CB or PB donor samples in experiment 1213 and 1, 2, or 3 of 4 donor samples in experiment 714). The critical observation for the purposes of identifying candidate genes was that in both experiments CB and PB monocytes differentially expressed many transcripts. The largest differences in expression were in transcripts included in the data on Lines 7 and 13; these gene products were consistently expressed only in CB or PB monocytes respectively. Transcripts included on lines 7, along with those represented on lines 8, 10, 11, and, more remotely, 12 could represented potential candidate genes contributing to the enhanced neuroprotective activity of CB monocytes. Transcripts included on lines 13 and 14 are not candidates. RMA provided quantitative information about the magnitude of differentially gene expression for each transcript. We mined the RMA expression data to identify over expressed CB monocyte transcripts that encode secreted proteins. Such proteins could account for ability of CB-CD14^+^ monocytes to protect brain neurons through the transwell membrane. We defined candidates as genes that (1) encoded secreted proteins or proteins that directly synthesized secreted products, (2) were over expressed in CB monocytes relative to PB monocytes in both microarray experiments, and (3) were differentially expressed in confirmatory quantitative PCR analysis using RNA from additional donors. This screen minimized the likelihood of variations in expression arising from differences in donor characteristics or methods used to purify CD14^+^ monocytes for RNA extraction. Seven candidates emerged from this analysis [Table 1].

**Supporting Figure Legend:**

**S1 Fig:** (A) Schematic diagram of organotypic brain slice culture in transwell system. (B) A representative CFSE-stained (green) CBMNC cells on a tissue slice after 72 hours. Red staining in top micrograph is from anti-human nuclei antibody staining, confirming that CFSE stained cell is of human origin. The cell shown had grown in size, put out numerous projections, and appeared to be highly activated. We used DAPI (blue) to stain the cell nuclei. Two different confocal planes (z-axis) of the same area are shown in lower two panels.

**S2 Fig:** Immunofluorescence images of CB-CD14^+^ and PB-CD14^+^ cells. Expression of TSP1, MMP9 and CHI3L1 is shown in red. CD14 expression is shown in green. Marge of both channels are also shown.

**S3 Fig**: Protein expression analysis of CB-CD14^+^ and PB-CD14^+^ cells. a) Lane 1-3, represent three different samples (n=3) of CB-CD14^+^ cells and Lane 4-6 represent, three different samples (n=3) of PB-CD14^+^ cells. B) Lane 1-2, represent two different samples (n=2) of CB-CD14^+^ cells and Lane 3-4 represent, two different samples (n=2) of PB-CD14^+^ cells. GAPDH was used as loading control. Quantitative expression of each proteins is shown in the table. No statistical significance (p< 0.05) was found in either cases.
