# Supplementary material for: Human umbilical cord blood monocytes, but not adult blood monocytes, rescue brain cells from hypoxic-ischemic injury: Mechanistic and therapeutic implications
Source: PLoS One. 2019 Sep 4;14(9):e0218906. doi: 10.1371/journal.pone.0218906 (PMC6726370; doi:10.1371/journal.pone.0218906)
Supplement: S1 Table — (DOCX) [file pone.0218906.s005.docx]

**S1 Table. Immunological reagents**

| **Name** | **Host** | **Source** | **Catalog#** | **Dilution used** |
| --- | --- | --- | --- | --- |
| ***Primary antibodies for IHC*** | | | | |
| GFAP | Chicken | Abcam, USA | ab4674 | 1:500 |
| NeuN | Mouse | Millipore, USA | MAB377 | 1:200 |
| Iba1 | Rabbit | Wako, USA | 01919741 | 1:1000 |
| ***Secondary antibodies for IHC*** | | | | |
| Anti-chicken Alexa Fluor 647 | Donkey | Jackson ImmunoResearch Laboratories, USA | 703-606-155 | 1:500 |
| Anti-rabbit Alexa Fluor 647 | Donkey | Life Technology, USA | A-31573 | 1:500 |
| Anti-rabbit Alexa Fluor 568 | Donkey | Life Technology, USA | A10042 | 1:500 |
| Anti-goat Alexa Fluor 568 | Donkey | Life Technology, USA | A-11057 | 1:500 |
| Anti-mouse Alexa Fluor 488 | Donkey | Life Technology, USA | A-21202 | 1:500 |
| ***Primary antibodies for WB*** | | | | |
| MMP9 | Rabbit | Abcam, USA | ab76003 | 1:10000 |
| Tsp1 | Rabbit | Abcam, USA | ab85762 | 1:1000 |
| CHI3L1 | Rabbit | Abcam, USA | ab77528 | 1:1000 |
| IL-10 | Rabbit | Abcam, USA | ab133575 | 1:1000 |
| Inhibin-A | Rabbit | Abcam, USA | ab128958 | 1:1000 |
| GAPDH | Rabbit | Cell Signaling Tech, USA | 5174 | 1:1000 |
| ***Secondary antibody for WB*** | | | | |
| Anti-rabbit HRP | Goat | Abcam, USA | ab6721 | 1:5000 |
|  |  |  |  |  |
