# Supplementary material for: Human umbilical cord blood monocytes, but not adult blood monocytes, rescue brain cells from hypoxic-ischemic injury: Mechanistic and therapeutic implications
Source: PLoS One. 2019 Sep 4;14(9):e0218906. doi: 10.1371/journal.pone.0218906 (PMC6726370; doi:10.1371/journal.pone.0218906)
Supplement: S2 Table — (DOCX) [file pone.0218906.s006.docx]

**S2 Table. Donor characterization and methods used to purify CD14^+^ monocytes from donor samples for subsequent microarray analysis.**

| Experiment | Source | Gender | Ethnicity | Hours* | Method^#^ | Purity** |
| --- | --- | --- | --- | --- | --- | --- |
| 1213 | PB-1 | Male | Caucasian | Fresh | 1 | 92 |
| 1213 | PB-2 | Male | Asian Indian | Fresh | 1 | 85 |
| 1213 | PB-4 | Male | Asian Indian | Fresh | 1 | 95 |
| 1213 | CB-A | Male | Caucasian | 18 | 1 | 87 |
| 1213 | CB-B | Male | Asian Indian | 15 | 1 | 92 |
| 1213 | CB-C | Male | Asian Indian | 9 | 1 | 91 |
|  |  |  |  |  |  |  |
| 714 | CB-1 | Male | Caucasian | 21 | 2 | 99.4 |
| 714 | CB-2 | Female | African American | 16 | 2 | 97.5 |
| 714 | CB-3 | Male | Caucasian | 13 | 2 | 97.4 |
| 714 | CB-4 | Male | Caucasian | 10 | 2 | 99 |
| 714 | PB-2 | Female | Asian Indian | Fresh | 2 | 99 |
| 714 | PB-4 | Male | Caucasian | Fresh | 2 | 97.9 |
| 714 | PB-5 | Male | Caucasian | Fresh | 2 | 99 |
| 714 | PB-6 | Male | Asian Indian | Fresh | 2 | 99.2 |

* Hours after collection of CB sample when isolation of CB-CD14^+^ cells was initiated.

**Percent of total population expressing CD14 determined by flow cytometry as previously described.

# Method 1: MNC fractions were prepared by centrifugation on Ficoll, treated with NH_4_Cl to remove erythrocytes, and CD14^+^ cells were immunomagnetically selected using Easysep [Stem Cell Technologies, Vancouver BC] as described by Saha et al.

Method 2: After NH4Cl lysis, MNC preparations were incubated on ice with PeCy7-mouse anti–human CD14 (BD catalog 562698), FITC-mouse anti–human CD3 (BD catalog 555339), and FITC-mouse anti–human CD235a (BD catalog 559943) antibodies. Cell suspensions were then flow sorted twice; for each sample an initial enrichment sort was followed by a purity sort to yield aCD14^+^CD235a^–^CD3^–^ population. Cells were maintained at 0°C–4°C during all procedures, including flow sorting.
