# Supplementary material for: Human umbilical cord blood monocytes, but not adult blood monocytes, rescue brain cells from hypoxic-ischemic injury: Mechanistic and therapeutic implications
Source: PLoS One. 2019 Sep 4;14(9):e0218906. doi: 10.1371/journal.pone.0218906 (PMC6726370; doi:10.1371/journal.pone.0218906)
Supplement: S3 Table — (DOCX) [file pone.0218906.s007.docx]

**S3 Table. Summary of MAS5 analysis of microarray results**

|  |  |  |  |  |  |  |
| --- | --- | --- | --- | --- | --- | --- |
|  |  |  | **1213** |  | **714** |  |
|  | **# CB samples** |  | 3 |  | 4 |  |
|  | **# PB samples** |  | 3 |  | 4 |  |
|  |  |  |  |  |  |  |
|  |  |  |  |  |  |  |
|  |  |  | Probeset # | | |  |
| 1 | Expressed by all CB samples |  | 18553 |  | 18514 |  |
| 2 | Not expressed by any CB samples |  | 27858 |  | 25919 |  |
| 3 | Expressed by all PB samples |  | 20275 |  | 19129 |  |
| 4 | Not expressed by any PB samples |  | 27188 |  | 25538 |  |
| 5 | Not expressed by any CB or PB samples |  | 24656 |  | 23014 |  |
| 6 | Expressed by all CB and PB samples |  | 17347 |  | 17140 |  |
|  |  |  |  |  |  |  |
| 7 | Expressed by all CB and no PB samples |  | 204 |  | 52 |  |
| 8 | Expressed by all CB and some PB samples |  | 1002 |  | 1322 |  |
|  |  |  |  |  |  |  |
| 9 | Expressed by some CB samples |  | 7212 |  | 10242 |  |
| 10 | Expressed by some CB and no PB |  | 2328 |  | 2472 |  |
| 11 | Expressed by some CB and some PB samples |  | 3482 |  | 5872 |  |
|  |  |  |  |  |  |  |
| 12 | Expressed by some CB and all PB |  | 2454 |  | 1899 |  |
| 13 | Expressed by some PB and no CB samples |  | 2328 |  | 2815 |  |
| 14 | Expressed by all PB & no CB samples |  | 474 |  | 90 |  |
| 15 | Expressed by some PB |  | 3730 |  | 10008 |  |
